# Lower Satisfaction and Inferior Outcomes Associated With Delayed Surgery for Chronic Quadriceps Tendon Ruptures: A Systematic Review

**DOI:** 10.1002/ars2.70004

**Published:** 2026-06-12

**Authors:** Matthew R. Bryan, Michael Mazzucco, Arsen Omurzakov, Alex E. White, Prashanth Kumar, Tyler Uppstrom, Nicolas Pascual‐Leone, Stephen O’Brien, Samuel A. Taylor

**Affiliations:** ^1^ Rothman Orthopaedic Institute Philadelphia Pennsylvania U.S.A.; ^2^ Weill Cornell Medical College New York New York U.S.A.; ^3^ Department of Sports Medicine Hospital for Special Surgery New York New York U.S.A.; ^4^ Columbia University Vagelos College of Physicians and Surgeons New York New York U.S.A.

## Abstract

**Purpose:**

To evaluate the literature regarding the efficacy of various repair and reconstruction surgical techniques for chronic quadriceps tendon ruptures (QTRs) in native knees and to assess their associated outcomes.

**Methods:**

A comprehensive literature search of the EMBASE, PubMed/MEDLINE, and Cochrane databases was conducted from October 2023 to November 2024 in accordance with Preferred Reporting Items for Systematic Reviews and Meta‐Analyses guidelines. Clinical studies that analyzed outcomes following surgical intervention of chronic QTRs with Level IV or greater evidence were included. We excluded case reports and QTRs in the setting of total knee arthroplasty. Quality assessment was conducted using Methodological Index for Non‐Randomized Studies criteria.

**Results:**

Ten studies published between 1981 and 2022 were included. Described techniques included direct tendon repair, lengthening procedures, and various graft and mesh augmentations. Reported failure rates ranged from 0 to 33%. Extensor lag remained a common reported complication (5 of 10 studies).

**Conclusions:**

The current body of literature on the surgical management of chronic QTRs is characterized by significant heterogeneity in surgical technique and a predominantly low quality of evidence. The various surgical techniques described suffer from limited follow‐up and inconsistently reported outcome measures.

**Level of Evidence:**

Level IV, systematic review of Level III and IV studies.

## Graphical abstract

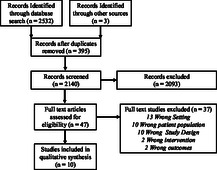

Quadriceps tendon ruptures (QTRs) are debilitating injuries that disproportionately affect middle‐aged male individuals, with a notable incidence of 1.37 patients per 100,000 persons annually.^1^ These injuries often cause substantial disability and present complex treatment challenges especially when surgical intervention is delayed or a repair fails. In particular, QTRs are most common in individuals older than 40 years and are frequently associated with high‐energy trauma in patients who have systemic conditions like diabetes and rheumatoid arthritis.^2^, ^3^, ^4^ Chronic QTRs are marked by loss of the tendon's multilayered organization, leading to tendon collagen fiber retraction.^5^ These injuries can severely impact mobility and quality of life, which underscores the necessity for prompt and accurate diagnosis and management.^6^, ^7^ In contrast to acute ruptures, which are often managed by primary repair, chronic ruptures and those of failed repair are typically complicated by fibrotic retraction and gap formation and are often not amenable to direct repair alone.^8^, ^9^, ^10^ In this review, we define chronic injuries as those greater than or equal to 3 weeks from injury to surgery. This definition is based on current best practices, which recommend repairing QTRs within 3 weeks, given that the cumulative evidence suggests that outcomes decline with surgical intervention after this time point.^9^

Chronic QTRs result in complex tissue damage and typically require more extensive surgical intervention when compared with acute injuries.^11^, ^12^ In select cases, repair may be possible; however, tendon lengthening and graft augmentation are often necessary. Several autograft and allograft options have been described in the literature, with varying rates of success.^13^, ^14^, ^15^, ^16^, ^17^, ^18^, ^19^ Despite the variety of surgical techniques described in the literature, there is a lack of high‐quality comparative studies on the surgical management of chronic QTRs and the optimal surgical technique is not well established.^20^, ^21^ Therefore, the purpose of this systematic review was to evaluate the literature regarding the efficacy of various repair and reconstruction surgical techniques for chronic QTRs in native knees and to assess their associated outcomes. We hypothesized that the techniques described for the management of chronic QTRs would exhibit heterogeneity and that the studies evaluating these techniques would predominantly be of low‐level evidence with limited follow‐up.

## METHODS

### Eligibility Criteria

All studies with Level IV or greater level of evidence (LOE) that described outcomes or complications following surgical management of chronic QTRs were included. Case reports of chronic QTRs have been previously summarized and were excluded from this review.^7^ Based on the authors’ language proficiency, only English‐language articles were eligible. Reviews, opinions, and animal, biomechanics, computational, and cadaveric studies were excluded. Studies that included acute QTRs only or did not separate outcomes based on chronicity of the injury were excluded. In this review, “chronic” was defined as greater than or equal to 3 weeks from injury to surgery or injuries explicitly described as “chronic” in the included study, where time from injury to surgery was otherwise not reported. Studies that examined QTRs in the setting of total knee arthroplasty and revision were also excluded.

### Search Strategy

This systematic review was conducted according to the standards of the Preferred Reporting Items for Systematic Reviews and Meta‐Analyses (PRISMA). From October 2023 to November 2024, the EMBASE, PubMed/MEDLINE, and Cochrane databases were queried. Queries included combinations of key search terms including “quadriceps tendon,” “quadriceps muscle,” “quadriceps insufficiency,” “tendon injury,” “repair,” “reconstruction,” “surgical technique,” “chronic tear,” and “chronic rupture.” Specific search methodology can be found in Supporting Information.

### Study Selection

The initial database search was performed by 2 authors (M.B., M.M.). Following this, 3 independent reviewers (M.B., M.M., A.O.) screened all titles and abstracts for relevance. Two of the same independent reviewers conducted full‐text screening for final inclusion (M.B., M.M.). A fourth reviewer (A.W.) was available to settle any conflicts for study inclusion. Reference sections of selected studies were reviewed to identify additional pertinent research for potential inclusion.

### Data Extraction

Data extraction was carried out independently by 3 authors (M.B., A.O., P.K.) using Covidence, a web‐based tool designed to streamline systematic reviews. Discrepancies in data extraction were resolved through discussion and consensus between the authors. Any remaining disagreements were arbitrated by a third author (A.W.). The extracted data included baseline publication information (journal, location, the year of publication) and demographic information (number of patients, gender distribution, patient comorbidities, presenting symptoms and mechanisms of injury, time to follow‐up, and surgical techniques). Primary outcomes were extracted, including patient‐reported outcome measures (PROMs) (Lysholm scores and IKDC scores), functional outcomes (range of motion [ROM] and development of extensor lag), and surgical failures and complications. We defined failure as any complication requiring revision surgery, which included chronic or recurring infection and rerupture.

### Methodological Quality Assessment

The assessment of methodological quality was independently conducted by 2 authors (A.O., P.K.) using the Methodological Index for Non‐Randomized Studies (MINORS), a validated tool designed to assess the methodological quality of nonrandomized studies. It includes 12 items, each scored from 0 to 2 (0—not reported, 1—reported but inadequately, 2—reported adequately), covering factors such as the clarity of the aim, inclusion of consecutive patients, prospective data collection, endpoints appropriate to the aim of the study, unbiased assessment of the study endpoint, follow‐up period appropriate to the aim, loss to follow‐up less than 5%, and prospective calculation of the study size. The total score ranges from 0 to 24 for noncomparative studies and from 0 to 32 for comparative studies. The mean MINORS score for this review was 13.9, indicating moderate quality. MINORS scores for each study are provided in Table 1, and detailed MINORS assessment is provided in Table S1.

**TABLE 1:** Characteristics of Included Studies

Author	Year	LOE	Number of Patients or Knees*	Percent Male (%)	Surgical Technique(s)	Follow‐Up Mean (mo)	Time to Surgery (wks; mean [range])	Most Common Comorbidities	MINORS Score
Rocha de Faria^22^	2022	IV	9	11.11	Modified Pulvertaft on Weave Technique	26	[9‐39]	CKD, HTN, and DM	16
Mahoney^23^	2021	IV	6	0	Codivilla V–Y Lengthening for Tendon Extension and LARS Artificial Ligament for Augmentation	NR	[6‐26]	RA, DM, CKD, and Steroid Abuse	12
Malta^24^	2014	III	11	66.67	Reinsertion With Marlex Mesh; Reinsertion With Hamstring Tendon Augmentation; Direct Reinsertion Without Augmentation	6	4.2	CKD, HTN, Glomerulonephritis, and Hepatitis C	20
Popov^25^	2013	III	29 (6)	3.45	Codivilla V‐Y Lengthening	42	NR	CKD, DM, and Obesity	16
Wilkins^26^	2010	IV	7 (3)†	28.57	Suture Placement for Reapproximation and Augmentation with Acellular Human Dermal Matrix (AHDM)	25	NR	NR	14
Siwek^27^	1981	III	67 (6)	13.43	End‐to‐End Suture With or Without Bunnell Pull‐Out Wire; Codivilla V‐Y in One Case	NR	NR	Gouty Arthritis, Psoriatic Arthritis, DM, and RA	16
Wenzl^28^	2004	III	36 (3)	94.30	Direct Repair With Transosseous Suture to the Patella	55	12‐52	None	18
Rizio^29^	2008	IV	3	33	Codivilla V‐Y Lengthening & Direct Repair With Transosseous Suture to the Patella	24	32 [20‐56]	Obesity, HTN, and Chronic LBP	6
Oni^30^	1985	IV	5	40	Vastus Lateralis Graft	NR	NR	DM	3
Rougraff^31^	1996	III	41 (11)	84.13	Direct Repair (Tendon to Tendon or Tendon to Patella With and Without Wire Augmentation)	67.2	13.2	NR	18

CKD, chronic kidney disease; DM, diabetes mellitus; HTN, hypertension; LARS, ligament augmentation and reconstruction system; LBP, low back pain; LOE, level of evidence; MINORS, Methodological Index for Non‐Randomized Studies; mo, month; NR, not reported, RA, rheumatoid arthritis; TKA, total knee arthroplasty.

* Total number of patients or knees (if different) in the study (number of chronic cases in parentheses).

† 7 cases of chronic QTR, 3 in native knees & 4 post‐TKA.

## RESULTS

**FIGURE 1:**
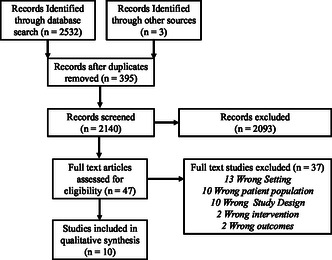
PRISMA (Preferred Reporting Items for Systematic Meta‐Analyses) flow chart for article inclusion.

A total of 2535 studies were identified through search of the EMBASE, PubMed/MEDLINE, and Cochrane databases and reference sections of relevant studies. A total of 395 duplicate publications were removed, resulting in 2140 unique articles. Subsequently, 2093 articles were excluded based on abstract and title screening in accordance with our exclusion criteria. The remaining 47 studies were retrieved for full‐text assessment, and 37 studies were further excluded due to wrong setting (n = 13), wrong study design (n = 10), wrong patient population (n = 10), wrong intervention (n = 2), and wrong outcomes (n = 2). Ten studies met the inclusion criteria and were included in this review (Figure 1).

### Study Characteristics

Two studies^22^, ^23^ were published in the 2020s, 3 studies^24^, ^25^, ^26^ were published in the 2010s, 2^28^, ^29^ were published in the 2000s, 1^31^ was published in the 1990s, and 2^27^, ^30^ were published in the 1980s. Sample size ranged from 3 to 67 for total (acute and chronic) patients and 3 to 13 for patients with chronic QTRs across the 10 studies. A total of 63 chronic QTRs were included in this review. Studies varied widely in the percentage of male patients (range 0‐94.3%). Five studies^24^, ^25^, ^27^, ^28^, ^31^ had an LOE of III, and 5 studies^22^, ^23^, ^26^, ^29^, ^30^ had an LOE of IV. Three studies^23^, ^27^, ^30^ did not report mean follow‐up, 1 study^24^ reported mean follow‐up of 6 months, and the remaining 6 studies^22^, ^25^, ^26^, ^28^, ^29^, ^31^ reported mean follow‐up greater than or equal to 24 months (range 6 months to 67.2 months). Four^25^, ^26^, ^27^, ^30^ of the 10 studies did not quantitatively report time from injury to surgery. Among the remaining 6 studies,^22^, ^23^, ^24^, ^28^, ^29^, ^31^ time from injury to surgery ranged from 6 to 56 weeks. Commonly reported medical comorbidities included obesity, diabetes mellitus, chronic kidney disease, and rheumatoid arthritis. Importantly, 1 study^24^ included only patients with end‐stage renal disease. Study characteristics and baseline demographic information are summarized in Table 1.

### Surgical Techniques

Various surgical techniques were evaluated across studies, including primary repair, tendon lengthening, and different forms of mesh and tendon graft augmentation. Four^24^, ^27^, ^28^, ^31^ of the 10 studies included patients who had undergone direct repair techniques in which the tendon was sutured directly to tendon or through transosseous drill holes in the patella without additional augmentation. Codivilla V‐Y lengthening of the tendon was described in 3 studies.^23^, ^25^, ^29^ Three studies^23^, ^24^, ^27^ described augmentation or reconstruction with a graft, including LARS artificial ligament augmentation (Mahoney et al.), hamstring tendon augmentation (Malta et al.), and fascial graft (Siwek and Rao). In 2 studies,^24^, ^26^ mesh material—marlex mesh (Malta et al.) or acellular dermal human matrix (Wilkins et al.)—was used to augment the repair. Oni^30^ described a technique for local vastus lateralis flap reinforcement, in which the aponeurotic portion of the vastus lateralis is pulled distally and medially and sutured to the distal tendon stump. Finally, Rocha de Faria et al.^22^ employed a modified Pulvertaft on Weave technique (PWT), which includes a semitendinosus tendon autograft augmentation sutured in a locking X formation over the remaining quadriceps tendon and transosseous fixation to the patella.

### Patient‐Reported Outcome Measures

Three studies^22^, ^25^, ^28^ included in this review evaluated PROMs, and only 1^22^ evaluated them before and after surgical intervention. IKDC scores, reported in de Faria's study, showed an increase from a preoperative mean of 30.3 ± 9.9 to a postoperative mean of 43.6 ± 12.3, although this change was not statistically significant (*P* = .07). They also reported a nonsignificant increase in Lysholm scores (preoperative 55.7 and postoperative 65.6, *P* = .21). Popov et al.^25^ and Wenzl et al.^28^ reported mean postoperative Lysholm scores of 81.4 and 92.5 respectively. The Wenzl et al.^28^ study reported a range of Lysholm scores from 46 to 100 and highlighted that 3 of the lowest scores (46, 50, and 81) were found in patients who underwent delayed rather than immediate surgical repair. Summary of PROMs are detailed in Table 2.

**TABLE 2:** Summary of Patient‐Reported Outcome Measures

Author	Pre‐Op IKDC, mean	Post‐Op IKDC, mean	Pre‐Op Lysholm Score, mean	Post‐Op Lysholm Score, mean	*P* Value
Popov^25^	‐	‐	‐	81.4	NR
Rocha de Faria^22^	30.3 ± 9.9	43.6 ± 12.3	55.7 ± 14.3	65.6 ± 19.1	IKDC = .07
					Lysholm = .21
Wenzl^28^	‐	‐	‐	92.5	NR

IKDC, International Knee Documentation Committee; NR, not reported.

### Failure Rates & Complications

Failure rates ranged from 0 to 33% among the studies that reported them. Of the studies that reported surgical failures requiring revision, only 5 of 48 knees required revision. Two of the 5 knees required revision due to recurrent or deep infection, 2 required revision due to rerupture, and 1 was not reported. In their series of 6 patients that underwent Codivilla V‐Y lengthening and artificial ligament augmentation, Mahoney et al.^23^ reported that 1 patient developed a superficial infection that resolved with a course of antibiotics and no additional complications. Similarly, Rougraff^31^ reported 1 case of superficial wound infection and 1 pulmonary embolism postoperatively. One study^25^ did not report complications separately for acute and chronic cases, but complications included quadriceps muscle atrophy, wire failure, and phlebothrombosis. Failure requiring revision and common postoperative complications are presented in Table 3.

**TABLE 3:** Described Techniques and Associated Failure Rates and Complications

Author	Technique	# Chronic QT Repairs	Failures Requiring Revision	Additional Complications
Rocha de Faria^22^	Modified Pulvertaft on Weave Technique.	9	NR	NR
Mahoney^23^	Codivilla V–Y Lengthening for Tendon Extension and LARS Artificial Ligament for Augmentation.	6	0	Superficial Wound Infection treated with Abx (1)
Malta^24^	Reinsertion of the Tendon With Marlex Mesh Augmentation.	3	0	None
	Reinsertion of the Tendon With Hamstring Tendon Augmentation.	6	0	None
	Direct Reinsertion Without Augmentation.	2	0	None
Popov^25^	Codivilla V‐Y Lengthening	6	1 (16.7%)	Atrophy of Quad Muscle (1), Tear of Wires (1), Phlebothrombosis (1), Avulsion of Patella (1), Contracture (1), Infection (1), Anchor Separation (1)†
Wilkins^26^	Suture Placement for Reapproximation and Augmentation With Acellular Human Dermal Matrix (AHDM)	3	1 (14.3%), Due to Recurrent Infection	None
Siwek^27^	End‐to‐End Suture, External Devices for Reinforcement, Pins‐And‐Wires, and Fascial Grafts.	6	NR	Hemarthrosis (1)
Wenzl^28^	Direct Repair With Transosseous Suture to the Patella	3	1 (33%), Due to Deep Infection	None
Rizio^29^	V‐Y Lengthening & Direct Repair With Transosseous Suture to the Patella	3	0	None
Oni^30^	Vastus Lateralis Graft	5	0	NR
Rougraff^31^	Direct Repair (Tendon to Tendon or Tendon to Patella With and Without Wire Augmentation)	11	2* (4.9%), Due to Rerupture	Superficial Wound Infection (1), Pulmonary Embolism (1)

Abx, antibiotics; NR, not reported; QT, quadriceps tendon.

* Number of failures among the entire cohort; not specified if failures were chronic cases.

† Complications for the entire cohort; not specified which occurred in chronic cases.

### ROM and Extensor Lag

Nine of the 10 studies^22^, ^23^, ^24^, ^26^, ^27^, ^28^, ^29^, ^30^, ^31^ reported postoperative ROM, while only 2 studies^22^, ^29^ reported both preoperative and postoperative ROM. Rocha de Faria's study^22^ reported a statistically significant improvement in full active ROM from a preoperative average of 79.5° ± 14.2° to 109° ± 15.4° postoperatively (*P* < .001). In their study utilizing V‐Y lengthening and direct repair, Rizio et al.^29^ found a mean preoperative passive flexion of 72°, which increased to 122° postoperatively. Mahoney's study,^23^ which also utilized Codivilla V‐Y lengthening and LARS ligament augmentation, reported a similar postoperative ROM of 108.75°. Siwek and Rao and Rougraff both^27^, ^31^ reported postoperative ROM for both delayed repair and immediate repair groups and found that immediate repair resulted in greater ROM compared with delayed repair. Siwek and Rao^27^ found that only 1 patient who underwent delayed repair achieved >90° of flexion, while all patients who underwent immediate repair achieved >120°. Rougraff^31^ found a mean ROM in delayed and immediate and repair groups of 119.4° and 124.6° respectively, which was not significantly different.

Of the studies that reported on extensor lag, 2 studies^22^, ^30^ with a total of 14 knees reported that zero patients developed extensor lag postoperatively. Malta et al.^24^ found a mean postoperative extensor lag of 2.8 ± 4.48 among 6 patients with end‐stage renal disease and 11 QTRs. Rocha de Faria et al.^22^ found statistically significant improvements in extensor lag following surgery, reporting a mean preoperative extensor lag of 46.5 ± 10.0 and postoperative extensor lag of 0 in all patients (*P* < .001). Like ROM, both Rougraff^31^ and Wenzl et al.^28^ found worse outcomes among patients who underwent delayed repair compared with immediate. Wenzl et al.^28^ found development of extensor lag >20° in only 2 patients, both of whom had delayed repair, while Rougraff^31^ found that 5/10 patients developed postoperative extensor lag in the delayed repair group, while 3/26 developed extensor lag in the immediate repair group (*P* < .05). Table 4 provides a summary of extensor lag findings in each study.

**TABLE 4:** Functional Outcomes

Author	Technique	Preop ROM (Average, Degrees)	Postop ROM (Average, Degrees)	Extensor Lag (Average, Degrees)
Rocha de Faria^22^	Modified Pulvertaft on Weave Technique.	79.5 ± 14.2	109.0 ± 15.4 (*P* < .001)	Preoperative: 46.5 ± 10.0, Postoperative: All Obtained 0; *P* < .001
Mahoney^23^	Codivilla V–Y Plasty for Tendon Extension and LARS Artificial Ligament for Augmentation.	NR	Full ROM in 4/6, 10° Extensor Lag in 1 Patient, 5‐110 in 1 Patient	Preoperative: NR, Postoperative: One Patient 10° Extension Lag at 6 wks
Malta^24^	Reinsertion of the Tendon With Marlex Mesh Augmentation.	NR	96.67	Preoperative: NR, Postoperative: 2.8 ± 4.48
	Reinsertion of the Tendon With Hamstring Tendon Augmentation.	NR	99.17	
	Direct Reinsertion Without Augmentation.	NR	95	
Popov^25^	Codivilla V‐Y Lengthening	NR	NR	NR
Wilkins^26^	Suture placement for Reapproximation and Augmentation With Acellular Human Dermal Matrix (AHDM)	NR	84.4 (R 0‐135)	Preoperative: NR, Post‐operative: Two Patients Had an Extensor Lag of 10, and One Patient Had an Extensor Lag of 30
Siwek^27^	End‐to‐End Suture, External Devices for Reinforcement, Pins‐And‐Wires and Fascial Grafts.	NR	Delayed: 1 Patient Achieved >90° of Flexion	NR
			Immediate: 0‐120 or More in All Knees	
Wenzl^28^	Direct Repair With Transosseous Suture to the Patella	NR	131.7 (R 50‐150)	Extensor Lag or Flexion Deficit >20 Was Only Found in Two Patients, Both Had Delayed Repair
Rizio^29^	V‐Y Lengthening & Direct Repair With Transosseous Suture to the Patella	72 Passive Flexion, Full Passive Extension	122 Passive Flexion, Full Passive Extension	NR
Oni^30^	Vastus Lateralis Graft	NR	>80 Active Flexion In 4/5	At 6 Months, 0 Patients Had Extensor Lag
Rougraff^31^	Direct Repair (Tendon to Tendon or Tendon to Patella With and Without Wire Augmentation)	NR	Delayed Repair ‐ 119.4 Immediate Repair ‐ 124.6 (Not Significantly Different)	5/10 Had Extensor Lag in the Delayed Group; 3/26 Had Extensor Lag in the Immediate Group (*P* < .05)

*Note*: Entire cohort average.

NR, not reported; ROM, range of motion.

### Patient Satisfaction and Additional Outcome Measures

Three studies^27^, ^28^, ^31^ reported subjective patient satisfaction postoperatively. Siwek and Rao^27^ found that all patients who underwent immediate repair graded their overall outcome as excellent, while 3 of 13 delayed repairs graded their outcome as unsatisfactory. Similarly, Wenzl et al.^28^ found that 3 patients were dissatisfied with their postoperative result, and all had undergone delayed repair. Finally, Rougraff^31^ found significantly higher satisfaction scores in the immediate group compared with the delayed group (Delayed group = 7.09; Immediate group = 9.07 [*P* < .05]). In terms of pain, Rocha de Faria et al.^22^ reported nonsignificantly lower pain on visual analog pain scale postoperatively compared with preoperatively (2.3 ± 2.4 points vs 1.8 ± 2.0 points, *P* < .07). Rougraff^31^ compared postoperative pain in immediate and delayed repair groups and found nonsignificantly lower pain in the immediate group (delayed = 7.27; immediate = 9.13, with 10 being no pain).

## DISCUSSION

The primary findings of this systematic review are that several surgical techniques exist for the management of chronic QTRs with a diversity of surgical outcomes reported with mostly short‐ to mid‐term follow‐up. While heterogeneity limits formal analysis, among the included studies, delayed repair or reconstruction was associated with worse postoperative outcomes, including worse pain scores, decreased ROM, increased development of an extensor lag, and lower satisfaction. In our review of 10 unique studies on chronic quad tendon injuries, described techniques included direct repair, Codivilla V‐Y lengthening procedures, and various reconstructive techniques utilizing allogeneic, autologous, and synthetic grafts. Additionally, of the studies that reported it, failure rates ranged from 0% to 33%. The existing literature on the surgical management of chronic quad tendon injuries is sparse and heterogeneous with limited long‐term outcomes.

Surgical timing is an important consideration in the management of QTRs. Four^25^, ^27^, ^28^, ^31^ of the 10 studies in this review included both immediate and delayed repairs of QTRs. One of those studies by Rougraff^31^ shows that patients who underwent delayed repair had significantly worse ROM, increased rates of postoperative extensor lag, lower satisfaction, greater need for an assistive walking device (e.g., cane), and greater difficulty ascending stairs. Similarly, Siwek and Rao^27^ and Wenzl et al.^28^ found that patients who underwent delayed repair had lower satisfaction, worse ROM, and higher rates of extensor lag. In line with these findings, the senior author of this review will often counsel patients that the goal in the chronic setting is to achieve greater than 90° of knee flexion postoperatively. In addition, Wenzl et al.^28^ reported increased pain severity and lower rates of return to work in their delayed repair group. In line with these studies, Scuderi reported a series of 20 cases where they showed worse postoperative outcomes in delayed repair compared with immediate repair and therefore recommended acute repair within 72 hours when possible.^32^ Similarly, Rougraff and Popov et al. recommended surgical intervention within 7 days of injury, while Siwek and Rao and Wenzl et al. recommended intervention within 14 days of injury.^25^, ^27^, ^28^, ^31^ A more recent review recommends intervention within 2‐3 weeks of injury.^9^ While failure was reported for only 5 of 63 patients included in this review, and follow‐up times were variable, the failure rates ranged from 0 to 33%. In comparison, a recent study of 245 primary quadriceps tendon repairs noted a failure rate of 11% with a median time from injury to surgery of 6 days.^33^ Thus, timely management of these injuries, when possible, may portend a more favorable outcome for these patients.

This systematic review highlights the heterogeneity of surgical techniques and limited long‐term data on the management of chronic QTRs. The heterogeneity in surgical technique suggests that there is a wide spectrum of tissue quality in these patients and that the individual procedures may be driven predominantly by interoperative findings. In general, QTRs in the chronic setting present a challenge due to significant scar formation and fibrosis, muscle contracture and atrophy, and large tendon gap formation due to tissue retraction. However, in certain instances, direct repair by suture or transosseous anchors may be pursued. Several studies in the present review report satisfactory outcomes following direct repair.^24^, ^28^, ^31^ Previous case reports have similarly reported satisfactory functional outcomes following direct repair of chronic QTRs.^34^, ^35^, ^36^ When there is tendon gap formation that does not allow for direct repair, lengthening techniques tend to be utilized, including Scuderi or Codivilla V‐Y advancement. The senior author prefers to perform a quadriceps tendon turndown technique in these difficult cases.^37^ The present review did not result in any Level IV or above studies that included the Scuderi technique, though 3 studies^23^, ^25^, ^29^ in this review (including 16 patients) described a Codivilla V‐Y lengthening technique. Rizio and Jarmon^29^ reported satisfactory postoperative ROM and high patient satisfaction, while Siwek and Rao^27^ reported an unsatisfactory result and achievement of only 90° of flexion in 1 delayed repair patient who underwent Codivilla V‐Y lengthening. Mahoney et al.^23^ reported full ROM at 6 weeks in 4/6 patients, zero cases of late extensor lag at 2 years' follow‐up, and zero failures utilizing Codivilla V‐Y lengthening with LARS artificial ligament augmentation. Additionally, the Codivilla V‐Y lengthening technique has been described in combination with other repair and reconstructive techniques with positive outcomes in numerous case reports.^10^, ^38^, ^39^

Various grafts have been utilized in the management of chronic QTRs. In the present review, there was no consensus graft choice utilized. The types of grafts described included a LARS artificial ligament, hamstring autograft, Marlex mesh, acellular human dermal allograft, and vastus lateralis autograft. Typically, these grafts were utilized as augmentation to an existing repair or lengthening procedure where there is tendon gap formation. Few comparative studies have assessed the outcomes of the various graft types for augmentation in chronic QTRs. In the present review, Malta et al.^24^ reported satisfactory outcomes in 6 patients with end‐stage renal disease with 11 chronic QTRs treated with Marlex mesh or hamstring tendon augmentation and found no significant differences in postoperative ROM between the 2 techniques (96.67 vs 99.17 for mesh and hamstring augments, respectively). There were zero reported failures or complications in either group. Several case reports have showed satisfactory outcomes utilizing hamstring tendon autografts^14^, ^15^ and Achilles and tibialis anterior allografts^19^, ^37^, ^40^ but were not included in this systematic review.

Strikingly, only 2 studies in the present review were published within the last 10 years. In the most contemporary study included in this review, Rocha de Faria et al.^22^ utilized a modified PWT. They found improvements, though nonsignificant, in PROMs and significant improvements in ROM and extensor lag postoperatively. Their finding that the involved limb showed equivalent early rate of torque development—a proxy for voluntary muscle recruitment and firing rate—suggests good functionality of the quadriceps muscle postoperatively as well. The PWT appears to result in similarly satisfactory outcomes when compared with traditional techniques. A quadriceps turndown technique that utilizes a reflected quadriceps tendon autograft has also recently been described.^41^ The paucity of recent, high‐LOE studies on the treatment of chronic QTRs is a clear void in the literature that should be addressed.

A recently published systematic review similarly describes the surgical treatment and outcomes for QTRs.^42^ The review includes more studies because it includes case reports; 19 of their 26 included studies are single case reports, which were excluded from our review. Despite this, our systematic review includes more cases of chronic QTR (63 vs 44) and more total knees (214). While their study does provide more background about causes for delayed intervention and reports on repair vs reconstruction—with significant limitations—our systematic review adds additional clinical value. First, our review attempts to remark on outcome differences based on time to surgery, with improved outcomes being noted with earlier intervention. Second, while limited due to heterogeneity, our study reports on patient reported outcomes and shows a decline in PROMs with increasing time to surgery. Lastly, our study adds additional contemporary techniques including the PWT and the quadriceps tendon turndown technique.

### Limitations

This review has several limitations. This systematic review included only LOE III and IV studies, and no prospective studies. Additionally, the heterogeneity in surgical techniques, patient demographics, and outcome measures across studies limits our ability to draw conclusions about superiority of specific surgical techniques and the generalizability of our findings. Only 1 study reported preoperative and postoperative PROMs; the ability to draw conclusions about PROMs following surgery for chronic QTRs is particularly weak. Small sample sizes across the included studies limits the power to detect meaningful differences between surgical techniques and timing of surgical intervention. Overall, heterogeneity and scarcity of data in the existing literature on chronic QTRs limits our ability to draw direct comparisons between the various treatment options.

## CONCLUSIONS

The current body of literature on the surgical management of chronic QTRs is characterized by significant heterogeneity in surgical technique and a predominantly low quality of evidence. The various surgical techniques described suffer from limited follow‐up and inconsistently reported outcome measures.

## SUPPORTING INFORMATION

Additional supporting information can be found online in the Supporting Information section.

## DISCLOSURES

The author (S.A.T.) declares the following financial interests/personal relationships, which may be considered as potential competing interests: S.A.T. reports a relationship with DJ Orthopedics that includes consulting or advisory. The other authors (M.R.B., M.M., A.O., A.E.W., P.K., T.U., N.P‐L., S.O.) declare that they have no known competing financial interests or personal relationships that could have appeared to influence the work reported in this article.

## Supporting information

Supplementary Material
