# Supplementary material for: Lower Satisfaction and Inferior Outcomes Associated With Delayed Surgery for Chronic Quadriceps Tendon Ruptures: A Systematic Review
Source: Arthrosc Sports Med Rehabil. 2026 Jun 12:e70004. Online ahead of print. doi: 10.1002/ars2.70004 (PMC13399835; doi:10.1002/ars2.70004)
Supplement: Supplementary file 1 — Supplementary Material [file ARS2-9999-e70004-s001.zip › Appendix 1.pdf]

Appendix 1: Search Strategy

“('quadriceps tendon' OR 'quadriceps muscle' OR 'quadriceps insufficiency') AND ('surgery'/syn OR 'tendon injury'/exp OR 'repair'/syn OR 'reconstruction'/syn OR 'augmentation'/syn OR 'revision'/syn OR 'surgical technique'/exp OR 'muscle flap'/exp) AND ('chronic' OR 'chronic disease'/exp OR 'tear'/syn OR 'insufficiency' OR 'rupture'/syn OR 'failed repair' OR 'extensor lag' OR 'treatment failure'/exp OR 'treatment outcome'/exp) NOT ('systematic review' OR 'expert opinions' OR 'conference abstract' OR 'review' OR 'level v' OR 'biomechanical' OR 'cadaveric' OR 'animal' OR 'acute quadriceps tendon surgery' OR 'intra-operative quadriceps rupture' OR 'anterior cruciate ligament reconstruction' OR 'anterior cruciate ligament repair' OR 'case report') AND [english]/lim
