# Supplementary material for: Lower Satisfaction and Inferior Outcomes Associated With Delayed Surgery for Chronic Quadriceps Tendon Ruptures: A Systematic Review
Source: Arthrosc Sports Med Rehabil. 2026 Jun 12:e70004. Online ahead of print. doi: 10.1002/ars2.70004 (PMC13399835; doi:10.1002/ars2.70004)
Supplement: Supplementary file 1 — Supplementary Material [file ARS2-9999-e70004-s001.zip › Supplemental Table 1.pdf]

Supplemental Table 1. MINORS assessment of Included Studies

Authors, years	Clear Aim	Inclusion of consecutive patients	Prospective collection of data	Endpoints appropriate to the aim of the study	Unbiased assessment of the study endpoint	Follow-up period appropriate to the aim of the study	Loss to follow up less than 5%	Prospective calculation of the study size	An adequate control group	Contemporary groups	Baseline equivalence of groups	Adequate statistical analyses:	Total
de Faria[23]	2	2	2	2	2	2	2	0	0	NA	NA	2	16
Mahoney[22]	2	2	2	2	2	0	2	0	0	NA	NA	0	12
Malta[24]	2	2	2	2	2	1	2	0	2	2	2	2	20
Popov, 2013	2	2	2	2	2	2	0	0	0	2	2	0	16
Wilkins, 2010	2	2	2	2	2	2	2	0	0	NA	NA	0	14
Popov[25]	2	2	2	2	2	0	2	0	0	2	2	0	16
Wilkins[26]	2	2	0	2	1	2	1	0	2	2	2	2	18
Siwek[31]	2	0	0	2	0	2	0	0	0	NA	NA	0	6
Wenzl[28]	2	0	0	1	0	0	0	0	0	NA	NA	0	3
Rizio[27]	2	2	0	2	1	2	1	0	2	2	2	2	18
